# Preference and Toxicity of Sulfoxaflor, Flupyradifurone, and Triflumezopyrim Bait against the Fire Ant *Solenopsis invicta* (Hymenoptera: Formicidae) and Their Efficacy under Field Conditions

**DOI:** 10.3390/insects15100813

**Published:** 2024-10-16

**Authors:** Jiefu Deng, Mei Yi, Mingrong Liang, Delong Tan, Weihui Bai, Cai Wang, Guiying Liu, Yijuan Xu, Yixiang Qi, Yongyue Lu, Lei Wang

**Affiliations:** 1 College of Plant Protection, South China Agricultural University, Guangzhou 510642, China; jefflonges@gmail.com (J.D.); ymydd1030@163.com (M.Y.); liangmr0321@connect.hku.hk (M.L.); b2451402768@163.com (W.B.); liuguiyingab@163.com (G.L.); xuyijuan@scau.edu.cn (Y.X.); qiyixiang19880922@163.com (Y.Q.); 2 Institute of Facility Agriculture, Guangdong Academy of Agricultural Sciences, Guangzhou 510642, China; tandelong@gdaas.cn; 3 College of Forestry and Landscape Architecture, South China Agricultural University, Guangzhou 510642, China; wangcai@scau.edu.cn

**Keywords:** red imported fire ant, sulfoxaflor, flupyradifurone, triflumezopyrim, poisonous bait, toxicity evaluation, control demonstration

## Abstract

The red imported fire ant *Solenopsis invicta* Buren (Hymenoptera: Formicidae) is native to South America; however, its introduction to other countries has caused serious biodiversity, agricultural, and public health problems. As toxic bait is an effective method to control fire ant populations, the aim of this study was to determine the most effective concentration of sulfoxaflor, flupyradifurone, and triflumezopyrim as ingredients for baits against *S. invicta* under laboratory and field conditions. Sulfoxaflor, flupyradifurone, and triflumezopyrim had no effect on the feeding behavior of the fire ants. However, they significantly reduced the climbing, walking, and arrest abilities of the fire ant workers after 10 days of treatment, and insecticides were horizontally transferred from workers to alates or larvae. Specifically, sulfoxaflor and triflumezopyrim at 0.05% concentration were the most effective in exterminating fire ants. Sulfoxaflor and triflumezopyrim are nonrepellent and effective insecticides against *S. invicta*.

## Simple Summary

The red imported fire ant *Solenopsis invicta* Buren poses a significant threat to biodiversity, agriculture, and public health in its introduced ranges. Chemicals are the main methods for *S. invicta* control. To explore more active ingredients for fire ant control, the toxicity and horizontal transfer effects of sulfoxaflor, triflumezopyrim, and flupyradifurone baits against S. invicta were determined and their field effects were assessed. Sulfoxaflor, flupyradifurone, and triflumezopyrim did not have affect the feeding behavior of the fire ants. However, they significantly reduced the climbing, walking, and arrest abilities of the fire ant workers after 10 days of treatment, and their toxicities were horizontally transferred from workers to alates or larvae. Specifically, the field trials showed that sulfoxaflor and triflumezopyrim at 0.05% concentration were the most effective in exterminating fire ants. The mortality rates of the mounds on Day 28 were 81.21% and 84.44% in 1.5- and 2.0-times 0.05% sulfoxaflor-treated plots, and the mortality rates of the mounds on Day 28 were 83.51% and 82.50% in 1.5- and 2.0-times 0.05% triflumezopyrim-treated plots.

## 1. Introduction

The red imported fire ant *Solenopsis invicta* Buren (Hymenoptera: Formicidae) is a notable invasive ant species that has been introduced to the United States of America, China, and several other countries and threatens the agriculture, public health, and biodiversity in the introduced regions [1,2,3]. Fire ants cause crop yield loss by destroying seeds and seedlings, disrupting insect and bird pollination, and interrupting the interactions between crops and honeydew-producing insects such as mealybugs and aphids [4,5,6,7,8]. People living in areas inhabited by fire ants often become stung by fire ants, which may lead to pain, swelling, redness, itching, anaphylactic shock, or even death [9,10]. Fire ants prey on various invertebrates and even small mammals, thereby reducing the biodiversity of the regions where they are introduced [11,12,13]. For example, more than 30% of native ants were eradicated in fire ant-invaded regions [14].

Bait is the most commonly used chemical method for controlling fire ant populations because they can eliminate entire fire ant colonies. The main ingredients of commercial baits are indoxacarb, hydramethylnon, and fipronil [15]. To prevent fire ant invasion in China, more than 50% of the 100 million Chinese yuan (CNY) allotted by the Chinese government for fire ant control in 2019 was spent on purchasing baits [16,17]. In the United States, the primary method of controlling S. invicta is chemical control, with commonly used baits with active ingredients of indoxacarb, hydramethylnon, and fipronil, which are more difficult to kill colonies intact [18,19,20]. On the other hand, in Japan, the pesticide considered to be the most promising agent for controlling *S. invicta* in the market is also fipronil [21]. In China, indoxacarb comprises 90% of the fire ant baits; fipronil is only used for indoor pests because of its high toxicity to bees. There is a risk of an increase in insecticide resistance among fire ants because baits with the same active ingredient have been applied in high doses in vast areas for a long period of time [22,23]. Hence, new and effective bait ingredients for managing *S. invicta* populations must be identified.

Some neonicotinoid insecticides have been registered as active ingredients in several commercial baits for *S. invicta* control [24]. Zhang et al. found that cycloxaprid is an effective neonicotinoid with slow-acting and nonrepellent properties against *S. invicta* workers [25]. Sulfoxaflor, triflumezopyrim, and flupyradifurone are fourth-generation neonicotinoids that act on the insect nicotinic acetylcholine receptor, though they demonstrate distinct modes of action [26,27,28]. They are effective in exterminating fire ant colonies even at very low concentrations [29,30,31] and are safer to nontarget organisms, especially bees, compared to the neonicotinoids developed previously [28,32]. Hence, sulfoxaflor, triflumezopyrim, and flupyradifurone have immense potential as effective ingredients for fire ant baits. In this study, the toxicity and horizontal transfer effects of sulfoxaflor, triflumezopyrim, and flupyradifurone baits against *S. invicta* were determined and their field efficacy were assessed. This study contributes to the development of baits that are effective in controlling fire ant populations, yet safe for nontarget organisms.

## 2. Materials and Methods

### 2.1. Insects and Chemicals

Fire ant colonies were collected from Huadu and Panyu counties of Guangzhou, China. All the fire ant colonies were polygyne. The fire ants were separated from mound soil using the water drip method, then transferred to a plastic container (Length×width×height: 400 mm × 300 mm × 150 mm) with a large ladle [33]. The inner wall of the rearing container was coated with a mixture of talcum powder and ethanol to prevent the fire ants from escaping [34]. The temperature in the feeding room was set at 25° and the humidity at 70%.

The fire ants were fed frozen locusts, which were purchased from a local pet food market, and 10% *w*/*w* sugar water, wherein the sugar was procured from a local store. Transform 22% sulfoxaflor SC (Dow AgroSciences, Beijing, China), Pyraxalt 10% triflumezopyrim SC (DuPont Crop Protection, Shanghai, China), and Sivanto 17% flupyradifurone SL (Bayer CropScience China, Shanghai, China) were purchased from a local pesticide store and were used for all experiments.

Each of the three insecticides was mixed with lard and dissolved in acetone; then, the solution was mixed with a corn grit carrier (Anhui Xifengshou Agricultural Science & Technology, Hefei, China) in a blender to product baits. The prepared baits were packed in Ziploc bags and stored at 4 °C until their subsequent use.

### 2.2. Feeding Bioassay

Before using the baits for toxicity determination, we used sugar water with insecticide to test the negative effects of sulfoxaflor, triflumezopyrim, and flupyradifurone on the feeding behavior of fire ants. A two-choice feeding bioassay was conducted to compare the amount of insecticide-treated sugar water consumed by the fire ants.

Two colonies were used for the experiment. Each colony was divided into eight sub-colonies with the same weight (>5 g), and each sub-colony contained workers and brood, so this experiment was repeated 16 times. After 24 h of water-only starvation treatment, each subcolony simultaneously received a 115 mL tube containing the control (10% *w*/*w* sugar water) and another 115 mL tube containing a single concentration of the insecticides (100 μg/mL or 50 μg/mL, these concentrations are based on the weight of the active ingredient of the product, the same below) in 10% *w*/*w* sugar water. The weight of each tube and subcolony were acquired after the removal of dead ants every day for 1 week. The daily sugar water consumption from each tube (milligram of sugar water per gram of ant per day) was also calculated for each subcolony to evaluate whether the insecticides affect ant feeding on sugar water. An identical sugar water tube was placed next to the subcolony and their natural evaporation was observed daily, and they were used to adjust the daily sugar water consumption.

### 2.3. Effects of Sulfoxaflor, Flupyradifurone, and Triflumezopyrim on the Survival of Different Castes/Developmental Stages of Fire Ants

Varying concentrations (100, 50, and 25 μg/mL) of sulfoxaflor and flupyradifurone were tested. Based on the results of a prior experiment, 200, 100, and 50 μg/mL of triflumezopyrim were used. Purified water was used as the control. This is a no-choice treatment.

One hundred twenty workers of varying sizes and 10 male or female alates were placed in a 250 mL bowl. Only one 1.5 mL centrifuge tube with 10% *w*/*w* sugar water containing one of the insecticides was placed in the bowl. The dead workers were removed, and healthy workers from the same colony were added to maintain the same number of workers every day to make sure there are enough workers to take care of the male or female alates. Counted the dead alates and recorded the daily mortality rate of the alates until all the alates had died. This treatment was conducted using two colonies with four replications per colonies for a total of eight replications.

Similarly, 120 workers of varying sizes and 10 larvae were placed in a 250 mL bowl. Only one 1.5 mL centrifuge tube with 10% *w*/*w* sugar water containing one of the insecticides was added to the bowl. The dead workers were removed, and healthy workers from the same colony were added to maintain the same number of workers every day. Counted the dead larvae and recorded the daily mortality rate of the larvae until all the larvae had died. The larvae were considered dead when their bodies had dried, and the workers were no longer interested in them [35]. This treatment was conducted using two colonies with four replications per colonies for a total of eight replications.

### 2.4. Toxicity of Sulfoxaflor, Flupyradifurone, and Triflumezopyrim Baits to Fire Ants and Their Effects on Fire Ant Behavior

This is a no-choice treatment. The baits were prepared following the methodology in the previous subsection outlined in insects and chemicals. A subcolony (5 g) with at least 80 larvae was placed in a plastic box and provided with purified water and an artificial nest was prepared using a Petri dish (diameter = 5 cm). After starvation for 24 h, varying concentrations of the baits (sulfoxaflor, flupyradifurone, and triflumezopyrim at 0.005%, 0.01%, 0.02%, or 0.05%) were provided. Indoxacarb bait at 0.1% concentration (Anhui Xifengshou Agricultural Science & Technology, Hefei, China) was used as the positive control because it is among the commonly used fire ant baits in China and corn grit carrier without insecticide was used as the negative control. The weight of each subcolony was acquired after removed dead ants and checked the mortality rate of the subcolony every 7 days. Each treatment was performed with three biological replications and two mechanical replications, for a total of eight replications.

After 10 days of treatment, the walking, climbing, and arrest abilities of the fire ants were assessed. For the walking ability test, 50 workers were placed on a paper and the workers that could not constantly walk for 5 s were considered to have lost their walking ability. The walking rate was calculated using the following equation:

Walking rate = (Number of walking workers/Total workers) × 100%

For the climbing ability test, 20 workers were placed in a cup for 10 s. A wooden stick was used to disturb the workers; workers that could not climb higher than 2 cm were considered to have lost their climbing ability. The climbing rate was calculated using the following equation:

Climbing rate = [1 − (Number of workers that cannot climb/Total workers)] × 100%

For the arrest ability test, 20 workers were placed in a cup for 10 s and the cup was then turned over. The number of workers that fell in 3 s was recorded, and the arrest rate was calculated using the following equation:

Arrest rate = [1 − (Number of falling workers/Total workers)] × 100%

This treatment was conducted using two colonies with four replications per colonies for a total of eight replications.

### 2.5. Preference of Fire Ants among Sulfoxaflor, Flupyradifurone, and Triflumezopyrim Baits

#### 2.5.1. Preference under Laboratory Conditions

**Figure 1:**
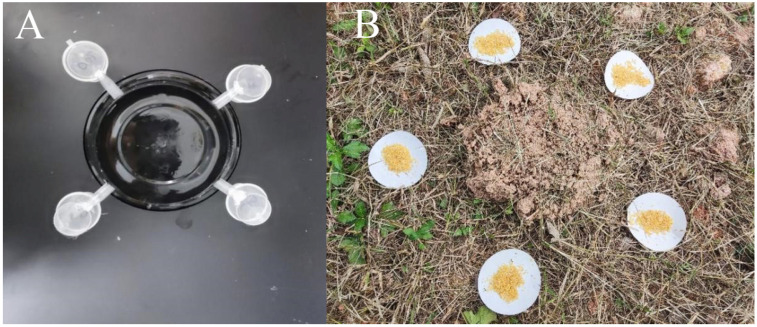
Preference of fire ants for sulfoxaflor, triflumezopyrim, and flupyradifurone baits. The equipment for (**A**) preference in laboratory; (**B**) the five filter paper containing baits with different concentration insecticide were placed around the mound.

A black bowl (diameter of the bottom = 12.5 cm, diameter of the mouth = 17.8 cm, height = 8 cm) was used as an artificial nest. Four Petri dishes (diameter = 5 cm; called foraging dishes) were connected by silicon tubes (diameter = 0.7 cm; length = 5 cm) to the artificial nest in different directions (Figure 1A).

A subcolony containing 1000 workers, 50 broods, and 5 female alates was placed in the artificial nest and starved for 24 h. 10 g of three different concentrations of the treatment baits and a control bait were randomly added to the four foraging dishes. The time when the first worker appeared on the dish was recorded. The number of workers on the foraging dish was recorded after 30 min. The weights of the baits removed by the fire ants were determined after 24 h. Natural water evaporation from the baits was also adjusted. The concentrations of the treatment baits with sulfoxaflor, flupyradifurone, and triflumezopyrim were 0.005%, 0.01%, and 0.02%. Indoxacarb bait (0.1%) was used as the control. The tests with sulfoxaflor, triflumezopyrim, and flupyradifurone baits were repeated nine times. The bioassay equipment was only used once.

#### 2.5.2. Preference under Field Conditions

Field bioassays were conducted on the South China Agricultural University campus, Guangzhou, China. Three different concentrations (0.005%, 0.01%, and 0.02%) of the treatment baits, a positive control bait (0.1% indoxacarb), and a negative control bait (corn grit carrier without insecticide) were applied on filter papers that were randomly placed 10 cm from and around fire ant mounds of fire ants (Figure 1B). The distances among the baits were the same, about 10–15 cm. The number of workers on the filter papers was recorded after 0.5, 1, 2, 3, and 4 h. The weight of the baits removed by the fire ants was measured after 4 h. The tests with sulfoxaflor, triflumezopyrim, and flupyradifurone baits were, respectively, repeated for different fire ant mounds, and 6 replicate trials were conducted for each agent separately.

### 2.6. Efficacy of Sulfoxaflor, Flupyradifurone, and Triflumezopyrim Baits in Single Mound Treatment

The field trials were conducted in a Camellia garden in Huadu county, Guangzhou, China. Both the field and lab colonies used in this study were polygyne. Based on the results of the laboratory experiment, the efficacy of the treatment baits at concentrations of 0.05%, 0.02%, 0.01%, and 0.005% was evaluated. An indoxacarb bait (0.1%) and corn grit carrier without insecticide were used as the positive and negative controls, respectively. Three plots were prepared for each treatment, and each 200 m^2^ plot had eight fire ant mounds. The diameter of each colony exceeded 10 cm. There are 42 plots prepared in this experiment in total, and baits were applied to each mound only once. A total of 20 g of the baits was distributed around the fire ant mounds (diameter = 10–30 cm) without disturbing the mounds and 30 g of the baits was applied if the diameter of the mound was over 30 cm. The distance between each plot was at least 5 m, and the fire ant colonies in the buffer zone were exterminated repeatedly by contact with 0.6% β-cypermethrin dust until all the fire ant colonies in the buffer zone inactive to eliminate their confounding effects on the experiment [36].

The activity of the fire ant mounds in each plot was checked 1 day before treatment application and 7, 14, 21, 28, and 35 days after the treatment. A fire ant mound was considered active if workers came out from the mound when disturbed. Meanwhile, the number of foraging workers in each treatment plot was trap counted using traps containing 5 mm thick sliced ham sausages 1 day before treatment application and 7, 14, 21, 28, and 35 days after the treatment. In total, 10 traps were placed per plot and trap counted after 30 min during each investigation.

At 35 days after the treatment, all the treated mounds were excavated completely to evaluate their mound level. Excavations need to be very thorough to determine if the queen is alive in the mounds. The level of each mound was classified in the following manner: grade 0, no caste of the colony survived; grade 1, either workers or brood of the colony survived; grade 2, all castes of the colony survived except the queen; and grade 3, all castes of the colony survived. All active mounds in the treatment and control plots were assigned a grade of 3 before treatment application. Consideration of a control plot can effectively correct for the effects of the environment during prolonged experiments, so the following equations (PN, PW, PC) are corrected by introducing data from the control plot.

The mortality rate of the fire ant mounds was calculated as:

PN (%) = [1 − (NO × NTi)/(NOi × NTO)] × 100

where PN is the mortality rate of fire ant mounds, NO is the number of active mounds before treatment application in the control plot, NTi is the number of active mounds post-treatment in the treatment plot, NOi is the number of active mounds post-treatment in the control plot, and NTO is the number of active mounds before treatment application in the treatment plot.

The reduction in the number of fire ant workers was calculated as:

PW (%) = [1 − (WO × WTI)/(WOI × WTO)] × 100

where PW is the reduction in the number of fire ant workers, WO is the average number of captured workers per trap before treatment application in the control plot, WTI is the average number of captured workers per trap post-treatment in the treatment plot, WOI is the average number of captured workers per trap post-treatment in the control plot, and WTO is the average number of captured workers per trap before treatment application in the treatment plot.

The reduction rate at the colony level was calculated as:

PC (%) = [1 − (CO × CTi)/(COi × CTO)] × 100

where PC is the control effect on the colony level, CO is the average colony level in the control plots before treatment application, CTi is the average colony level in the treatment plots 35 days post-treatment, COi is the average colony level in the control plots post-treatment, and CTO is the average colony level in the treatment plots before treatment application.

The comprehensive control effect of the baits against fire ants (P) was calculated as:

P = 0.3PN + 0.2PW + 0.5PC.

### 2.7. Efficacy of Sulfoxaflor, Flupyradifurone, and Triflumezopyrim Baits with Broadcast Application

To determine the recommended application rate of each treatment bait by broadcast application, a bait spreading test was performed at Wuzhipa Park in Shenzhen, China. Based on the results of the field trial of mound treatment, 0.05% concentration of the treatment baits was selected and an indoxacarb bait (0.1%) and corn grit carrier without insecticide were used as the positive and negative controls, respectively.

Based on the application rate, the experimental plots of the treatment baits were divided into 1.5- and 2.0-times sulfoxaflor-, flupyradifurone-, and triflumezopyrim-treated plots. The application dosage of 1.5- and 2.0-times sulfoxaflor-, flupyradifurone-, and triflumezopyrim-treated plots was calculated using the following equations:

Application dosage of 1.5-times sulfoxaflor-, flupyradifurone-, and triflumezopyrim-treated plots = Nm × 20 g/mound × 1.5

Application dosage of 2.0-times sulfoxaflor-, flupyradifurone-, and triflumezopyrim-treated plots = Nm × 20 g/mound × 2

where Nm is the number of active fire ant mounds in the plot.

The negative and positive controls only had 2.0-times application dosage plots. Each plot had more than seven mounds and an area of over 6000 m^2^. The bait spreading test was performed thrice in each treatment plot. The distance between each plot was at least 40 m, and the fire ant colonies in the buffer zone were exterminated repeatedly by contact with 0.6% β-cypermethrin dust until all the fire ant colonies in the buffer zone were inactived, to eliminate their confounding effects on the experiment.

A two-step method was employed in the experimental plots: the baits were scattered manually, and the baits were reapplied 14 days after the initial treatment application to completely exterminate the remaining fire ants in the mounds [37]. The activity of the fire ant mounds in each plot was checked 1 day before treatment application and 14 and 28 days after the treatment; the number of foraging workers in each experimental plot was trap counted using traps containing 5 mm thick sliced ham sausages 1 day before treatment application and 14 and 28 days after the treatment.

### 2.8. Data Analysis

Shapiro–Wilk and Levene’s tests were performed to test the normal distribution of the data and homogeneity of variance, respectively. Subsequently, one-way analysis of variance and Tukey’s test were applied for multiple comparisons of the data that satisfied the normal distribution and even variance conditions. Otherwise, nonparametric Kruskal–Wallis test and χ^2^ test was performed to compare the medians. Significant differences at *p* < 0.05 were subjected to Mann–Whitney test for pairwise comparisons. All statistical analyses were performed using SPSS 21.0 (SPSS, Chicago, IL, USA).

## 3. Results

### 3.1. Feeding Bioassay

**Figure 2:**
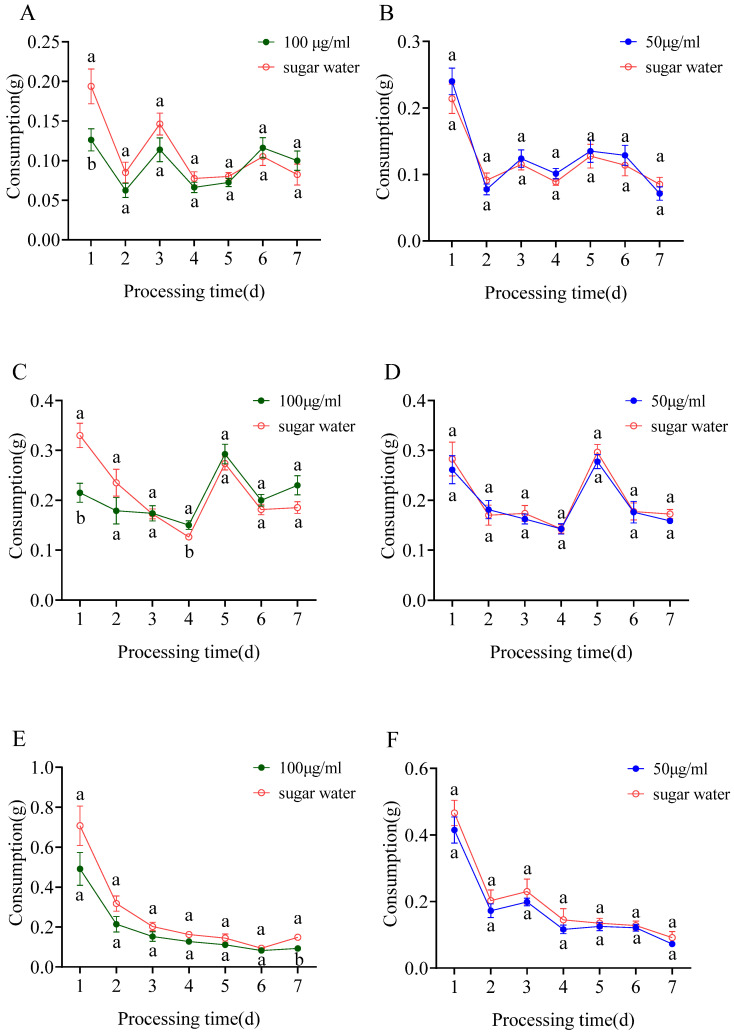
Effects of sulfoxaflor (**A**,**B**), flupyradifurone (**C**,**D**), and triflumezopyrim (**E**,**F**) at different concentrations on the food consumption of the fire ants for 7 days (means ± SE). The same letter at the same time in the figure indicates no significant difference by the independent samples *t* test (*p* > 0.05).

This experiment was repeated 16 times. The results of the bioassay revealed that the fire ants consumed less of the sugar water containing 100 μg/mL sulfoxaflor than the control on Day 1 of the experiment (t = −2.599, df = 14, *p* = 0.021); however, this difference was not significant from Days 2–7 (Figure 2A). There was no significant difference in the consumption of sugar water containing 50 μg/mL sulfoxaflor and sugar water consumed in the control group from Days 1–7 (Figure 2B).

The fire ants consumed less sugar water containing 100 μg/mL flupyradifurone than control on Day 1 (t = −3.683, df =14, *p* = 0.002); however, they consumed more sugar water containing 100 μg/mL flupyradifurone than the control on Day 4 (t = 2.297, df =14, *p* = 0.038). There was no significant difference between the consumption of the control and sugar water containing 100 μg/mL flupyradifurone from Days 2–7 (Figure 2C). There was no significant difference in consumption of sugar water containing 50 μg/mL flupyradifurone and sugar water consumed in the control group from Days 1–7 (Figure 2D).

The fire ants consumed less of the sugar water containing 100 μg/mL triflumezopyrim than control on Day 7 (t = −3.170, df =14, *p* = 0.007); however, this difference was not significant from Days 1–6 (Figure 2E). There was no significant difference in consumption of sugar water containing 50 μg/mL triflumezopyrim and sugar water consumed in the control group from Days 1–7 (Figure 2F).

### 3.2. Effects of Sulfoxaflor, Flupyradifurone, and Triflumezopyrim on the Survival of Different Castes/Developmental Stages of Fire Ants

Each of the castes was established using two colonies with four replications per colony for a total of eight replications. The mortality rate of the fire ants increased as the concentration of the treatment baits increased. The female alates died more slowly than the male alates and larvae after being fed the treatment baits.

**Figure 3:**
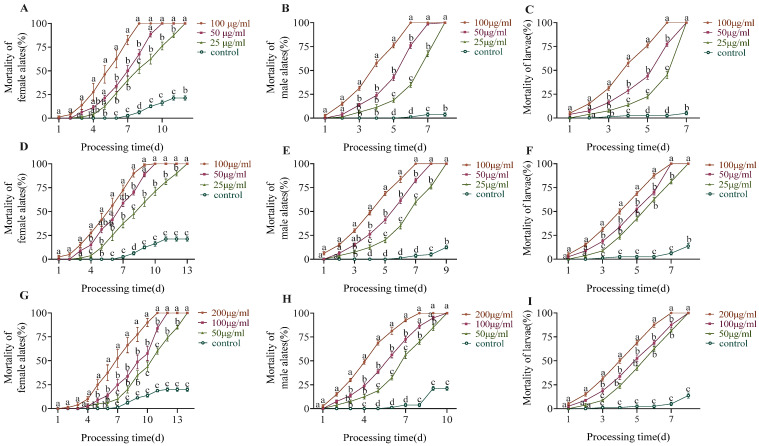
Effects of sulfoxaflor (**A**–**C**), flupyradifurone (**D**–**F**), and triflumezopyrim (**G**–**I**) at different concentrations on the survival of different developmental stages of fire ant for 14 days. The same letter at the same time in the figure indicates no significant difference by Kruskal–Wallis test (*p* > 0.05).

All the female alates that were fed 100 μg/mL sulfoxaflor died before Day 8, whereas all the male alates and larvae died before Day 7 (Figure 3A–C). After 8 days of treatment, the mortality rate of the female alates that were fed 100 μg/mL sulfoxaflor was significantly higher than that with other treatments (χ^2^ = 27.987, df = 3, *p* < 0.0001). After 6 days of treatment, the mortality rates of the male alates (χ^2^ = 30.038, df = 3, *p* < 0.0001) and larvae (χ^2^ = 29.885, df = 3, *p* < 0.0001) that were fed 100 μg sulfoxaflor were significantly higher than the other sulfoxaflor.

All the female alates that were fed 100 μg/mL flupyradifurone died before Day 10, whereas all the male alates and larvae died before Day 7 (Figure 3D–F). After 6 days of treatment, the mortality rate of the female alates that were fed 100 μg/mL flupyradifurone was significantly higher than that with other flupyradifurone treatments (χ^2^ = 28.092, df = 3, *p* < 0.0001). After 5 days of treatment, the mortality rates of the male alates (χ^2^ = 29.138, df = 3, *p* < 0.0001) and larvae (χ^2^ = 27.031, df = 3, *p* < 0.0001) that were fed 100 μg/mL flupyradifurone were significantly higher than the other flupyradifurone.

All the female alates that were fed 200 μg/mL triflumezopyrim died before Day 11, whereas all the male alates and larvae died before Day 8 (Figure 3G–I). After 5 days of treatment, the mortality rate of the female alates that were fed 200 μg/mL flupyradifurone was significantly higher than that with other treatments (χ^2^ = 25.434, df = 3, *p* < 0.0001). After 3 days of treatment, the mortality rate of the male alates that were fed 200 μg/mL triflumezopyrim was significantly higher than that with other treatments (χ^2^ = 27.974, df = 3, *p* < 0.0001). After 6 days of treatment, the mortality rate of the larvae that were fed 200 μg/mL triflumezopyrim was significantly higher than that with other treatments (χ^2^ = 27.314, df = 3, *p* < 0.0001).

### 3.3. Toxicity of Sulfoxaflor, Flupyradifurone, and Triflumezopyrim Baits on Fire Ants and Their Effects on Fire Ant Behavior

#### 3.3.1. Toxicity of Sulfoxaflor, Flupyradifurone, and Triflumezopyrim Baits to Fire Ants

In this experiment, each treatment was performed with three biological replications and two mechanical replications, for a total of eight replications. Table 1 shows the mortality of the fire ant workers treated with varying concentrations of sulfoxaflor, flupyradifurone, and triflumezopyrim. The mortality rate of the fire ant workers that were fed 0.05% sulfoxaflor bait did not differ from that of the ants that were fed 0.1% indoxacarb bait; however, the mortality rate was significantly higher than that of the ants that were fed the corn grit carrier without insecticide (χ^2^ = 34.345; df = 5; *p* < 0.001).

The mortality rate of the fire ant workers that were fed 0.05% flupyradifurone bait was significantly lower than that of the ants that were fed 0.1% indoxacarb bait; however, the mortality rate was significantly higher than that of the ants that were fed the corn grit carrier without insecticide (χ^2^ = 32.728; df = 5; *p* < 0.001).

Furthermore, the mortality rate of the fire ants that were fed 0.05% triflumezopyrim bait was significantly lower than that of the ants fed with 0.1% indoxacarb bait; however, the mortality rate was significantly higher than that of the ants that were fed corn grit carrier without insecticide (χ^2^ = 33.639; df = 5; *p* < 0.001).

#### 3.3.2. Effects of Sulfoxaflor, Flupyradifurone, and Triflumezopyrim Baits on the Behavior of Fire Ants

Table 2 shows the walking, climbing, and arrest rates of the workers treated with varying concentrations of the sulfoxaflor bait. After 10 days of treatment, the walking rates (F = 32.090; df = 4, 35; *p* < 0.001), climbing (F = 36.643; df = 4, 35; *p* < 0.001), and arrest (χ^2^ = 32.284; df = 4; *p* < 0.001) of the workers that were fed sulfoxaflor were significantly lower than those of the workers that were fed corn grit carrier without insecticide.

Table 3 shows the walking, climbing, and arrest rates of the workers treated with varying concentrations of the flupyradifurone bait. After 10 days of treatment, the walking (F = 19.242; df = 4, 35; *p* < 0.001), climbing (χ^2^ = 20.707; df = 4, 35; *p* < 0.001), and arrest (χ^2^ = 33.917; df = 4; *p* < 0.001) rates of the workers that were fed flupyradifurone were significantly lower than those of the workers that were fed the corn grit carrier without insecticide.

Table 4 shows the walking, climbing, and arrest rates of the workers treated with varying concentrations of the triflumezopyrim bait. After 10 days of treatment, the walking (χ^2^ = 23.049; df = 4; *p* < 0.001), climbing (F = 14.739; df = 4, 35; *p* < 0.001), and arrest (χ^2^ = 32.656; df = 4; *p* < 0.001) rate of the workers that were fed triflumezopyrim were significantly lower than those of the workers that were fed the corn grit carrier without insecticide.

### 3.4. Preference of Fire Ants among Sulfoxaflor, Flupyradifurone, and Triflumezopyrim Baits

#### 3.4.1. Preference under Laboratory Conditions

The discovery times of the sulfoxaflor, flupyradifurone, and triflumezopyrim baits at different concentrations by the fire ants and the weight of the removed baits are shown in Table 5. The discovery times of the corn grit carrier and the 0.02%, 0.01%, and 0.005% sulfoxaflor baits by the fire ants were 40.4 s, 75.3 s, 110.7 s, and 69.3 s, respectively. There was no significant difference between the discovery times of the sulfoxaflor baits and negative control (F = 0.590; df = 3, 36; *p* = 0.626). After 24 h, the weights of the removed corn grit carrier and the 0.02%, 0.01%, and 0.005% sulfoxaflor baits were 180.0, 121.1, 131.1, and 187.8 mg, respectively. There was no significant difference between the weights of the removed sulfoxaflor baits and negative control (F = 1.076; df = 3, 36; *p* = 0.373).

Moreover, the discovery times of the corn grit carrier and the 0.02%, 0.01%, and 0.005% flupyradifurone baits by the fire ants were 141.2, 254.9, 211.4, and 131.1 s, respectively. There was no significant difference between the discovery times of the flupyradifurone baits and negative control (χ^2^ = 0.874; df = 3; *p* = 0.832). After 24 h, the weights of the removed corn grit carrier and the 0.02%, 0.01%, and 0.005% flupyradifurone baits were 137.8, 13.3, 14.4, and 76.7 mg, respectively. The fire ants moved a lesser weight of the 0.02% and 0.01% flupyradifurone baits than that of the corn grit carrier (0.02% bait, U =14, *p* = 0.014; 0.01% bait, U = 14, *p* = 0.014). There was no significant difference between the weights of the removed 0.005% flupyradifurone bait and the corn grit carrier (U = 34; *p* = 0.565).

Lastly, the discovery times of the corn grit carrier and the 0.02%, 0.01%, and 0.005% triflumezopyrim baits by the fire ants were 109.6, 187.1, 126.8, and 168.1 s, respectively. There was no significant difference between the discovery times of the triflumezopyrim baits and negative control (F= 0.680; df = 3, 36; *p* = 0.571). After 24 h, the weights of the removed corn grit carrier and the 0.02%, 0.01%, and 0.005% triflumezopyrim baits were 163.3, 86.7, 120.0, and 110.5 mg, respectively. There was no significant difference between the weights of the removed triflumezopyrim baits and negative control (F = 0.778; df = 3, 36; *p* = 0.516).

#### 3.4.2. Preference under Field Conditions

Table 6 shows the discovery times of the three treatment baits by the fire ants and the weights of the removed baits. There was no significant difference between the discovery times of the sulfoxaflor baits and controls (F = 0.757; df = 4, 25; *p* = 0.563) and between the weights of the removed sulfoxaflor baits and controls at 4 h (χ^2^ = 2.943; df = 4; *p* = 0.567).

Furthermore, there was no significant difference between the discovery times of the flupyradifurone baits and controls (F = 0.751; df = 4, 25; *p* = 0.567) and between the weights of the removed flupyradifurone baits and controls at 4 h (χ^2^ = 5.632; df = 4; *p* = 0.228).

Lastly, there was no significant difference between the discovery times of the triflumezopyrim baits and controls (F = 0.157; df = 4, 25; *p* = 0.958) and between the weights of the removed triflumezopyrim baits and controls at 4 h (χ^2^ = 6.724; df = 4; *p* = 0.151).

### 3.5. Efficacy of Sulfoxaflor, Flupyradifurone, and Triflumezopyrim Baits in Single Mound Treatment

Table 7 shows the mortality rates on Day 35 of the fire ants treated with varying concentrations of the treatment baits. The mortality rate of the fire ants treated with 0.05% sulfoxaflor bait reached 91.67%, which was significantly higher than that of the fire ants treated with other sulfoxaflor concentrations; however, it was not significantly different from that of the mounds treated with 0.1% indoxacarb bait (χ^2^ = 16.541; df = 5; *p* = 0.005). The mortality rate of the fire ants treated with 0.05% flupyradifurone bait reached 54.17%, which was significantly higher than that of the ants treated with other flupyradifurone concentrations; however, it was significantly lower than that of the ants treated with 0.1% indoxacarb bait (χ^2^ = 16.730; df = 5; *p* = 0.005). The mortality rate of the fire ants treated with 0.05% triflumezopyrim reached 83.33%, which was not significantly different from the mortality rate of the mounds treated with 0.02% and 0.01% triflumezopyrim and 0.1% indoxacarb bait (F= 24.3; df = 5, 12; *p* < 0.0001). However, the mortality rate of the fire ants treated with 0.1% indoxacarb bait was significantly higher than that of the fire ants treated with 0.02%, 0.01%, and 0.005% triflumezopyrim baits (F= 24.3; df = 5, 12; *p* < 0.0001).

After the application of the treatment baits, mound scores were determined. A low mound score (0–3) means a low movement rate of the treated mounds, indicating favorable efficacy of the treatment baits in controlling fire ant populations. The mound score of the 0.05% sulfoxaflor-treated plots was 1.27; thus, new mounds did not appear around the treated mounds. This mound score was significantly lower than that of the 0.005% sulfoxaflor-treated plots, which was 2.20 (χ^2^ = 13.138; df = 5; *p* = 0.022). The mound score of the 0.05% flupyradifurone-treated plots was 2.30, indicating that new mounds did not appear around the treated mounds. This mound score was significantly lower than that of the 0.005% flupyradifurone-treated plots, which was 3.37 (χ^2^ = 12.924; df = 5; *p* = 0.017). The mound score of the 0.05% triflumezopyrim-treated plots was 1.47, indicating that new mounds did not appear around the treated mounds. This mound score was significantly lower than that of the 0.005% triflumezopyrim-treated plots, which was 2.87 (χ^2^ = 13.587; df = 5; *p* = 0.013).

The efficacy of the 0.05% sulfoxaflor bait in controlling fire ant colonies was 92.27%, which was significantly higher than that of the other sulfoxaflor bait concentrations; however, its efficacy was not significantly different from that of the 0.1% indoxacarb bait (χ^2^ = 16.660; df = 5; *p* = 0.005). The efficacy of 0.05% flupyradifurone bait in controlling fire ant colonies was 49.83%, which was not significantly different from that of 0.02% and 0.01% flupyradifurone baits; however, its efficacy was significantly lower than that of the 0.1% indoxacarb bait (χ^2^ = 15.691; df = 5; *p* = 0.008). The efficacy of the 0.05% triflumezopyrim bait in controlling fire ant colonies was 73.33%, which was not significantly different from that of the 0.02% triflumezopyrim bait; however, its efficacy was significantly lower than that of the 0.1% indoxacarb bait (χ^2^ = 16.387; df = 5; *p* = 0.006).

After 35 days of 0.05% sulfoxaflor bait treatment, the fire ant colony levels decreased to 0.23, which was significantly lower than that induced by the negative control (F = 171.116; df = 5, 12; *p* < 0.0001). After 0.05% triflumezopyrim bait treatment, the fire ant colony levels decreased to 1.36, which was significantly lower than that induced by the negative control (χ^2^ = 16.469; df = 5; *p* = 0.006). After 0.05% triflumezopyrim bait treatment, the fire ant colony levels decreased to 0.73, which was significantly lower than that induced by the negative control (F = 73.444; df = 5, 12; *p* < 0.0001).

The reduction rates of the foraging workers after 35 days of bait treatment are shown in Table 8. The reduction rate of the foraging workers increased as the treatment duration was prolonged.

The reduction rate of the foraging workers exposed to the 0.05% sulfoxaflor bait was 59.5%, which was not significantly different from that of the workers treated with other sulfoxaflor bait concentrations, 0.1% indoxacarb bait, and corn grit carrier; however, the reduction rate of the foraging workers treated with the 0.1% indoxacarb bait was significantly higher than that of the workers treated with 0.01% and 0.005% sulfoxaflor baits (F = 4.957; df = 5, 12; *p* = 0.011).

In addition, the reduction rate of the foraging workers exposed to the 0.05% flupyradifurone bait was 66.6%, which was not significantly different from that of foraging workers treated with other flupyradifurone bait concentrations and 0.1% indoxacarb bait (F = 8.776; df = 5, 12; *p* = 0.001).

Furthermore, the reduction rate of the foraging workers exposed to the 0.05% triflumezopyrim bait was 74.2%, which was not significantly different from that of the workers treated with other triflumezopyrim bait concentrations and 0.1% indoxacarb bait (χ^2^ = 13.070; df = 5; *p* = 0.023).

Table 9 shows that the comprehensive control effects of the 0.05% sulfoxaflor, 0.05% flupyradifurone, and 0.05% triflumezopyrim baits were 86.43%, 54.49%, and 77.18%, respectively, at 35 days post-treatment. The comprehensive control effect of the 0.05% sulfoxaflor bait did not differ significantly from that of the 0.1% indoxacarb bait, which was 96.09%; however, the comprehensive control effect of the 0.05% sulfoxaflor bait was significantly higher than that of 0.05% flupyradifurone (F = 72.014; df = 13, 28; *p* < 0.001). However, the comprehensive control effect of the 0.05% flupyradifurone bait was significantly lower than that of the 0.05% sulfoxaflor and 0.05% triflumezopyrim baits. The comprehensive control effect of the 0.05% triflumezopyrim bait did not differ significantly from that of 0.05% sulfoxaflor; however, it was significantly lower than that of the 0.1% indoxacarb bait. The comprehensive control effects of 0.02% sulfoxaflor, 0.02% triflumezopyrim, and 0.05% triflumezopyrim baits were, thus, not significantly different.

### 3.6. Efficacy of Sulfoxaflor, Flupyradifurone, and Triflumezopyrim Baits by Broadcast Application

As a result of the broadcast application of the treatment baits using the two-step control method, the mortality rate of the mounds and reduction rate of the foraging workers increased as the treatment dosages and duration increased (Table 10). The mortality rates of the mounds on Day 28 were 81.21% and 84.44% in 1.5- and 2.0-times 0.05% sulfoxaflor-treated plots, respectively. They did not differ significantly from that of 0.1% indoxacarb-treated and 2.0-times 0.05% triflumezopyrim-treated plots, which were 83.51% and 82.50%, respectively, but the mortality rates of the mounds were significantly higher than that of the mounds with other treatments (F = 80.391; df = 7, 16; *p* < 0.001). The mortality rates of the mounds in 1.5- and 2.0-times 0.05% flupyradifurone-treated plots were 52.80% and 52.08%, respectively.

Moreover, the reduction rates of the foraging workers on Day 28 were 71.85% and 80.96% in 1.5- and 2.0-times 0.05% sulfoxaflor-treated plots, respectively. The reduction rates of the foraging workers were significantly higher than that of the workers treated with the 0.1% indoxacarb bait at 53.71% (F = 58.355; df = 7, 16; *p* < 0.001). The reduction rates of the foraging workers were 71.85%, 62.30%, and 62.20% in 1.5-times 0.05% sulfoxaflor-treated, 0.05% flupyradifurone-treated plots, and 2.0-times 0.05% triflumezopyrim-treated plots, respectively; however, there was no significant difference among the three treatments.

## 4. Discussion

A good active ingredient of fire ant baits exhibits delayed action, is readily transferred among colonies, and is not repellent when combined with a bait carrier [38]. Some neonicotinoid insecticides, such as imidacloprid and thiamethoxam, have antifeeding effects at high concentrations; however, such effects disappear at low concentrations [39,40,41,42]. In our study, we found that 100, 50, and 25 μg/mL sulfoxaflor, flupyradifurone, and triflumezopyrim baits did not induce any negative effects on the feeding behavior of fire ants. Previous studies have shown that sulfoxaflor, flupyradifurone, and triflumezopyrim at low concentrations, such as those below 10 μg/mL, are nonrepellent [29,30,31]. When the treatment baits were combined with a corn grit bait carrier, sulfoxaflor and triflumezopyrim at 0.02%, 0.01%, and 0.005% concentrations did not alter the timing of food discovery and retrieval of the bait by ants. Although the food discovery time of the fire ants was not altered by 0.02%, 0.01%, and 0.005% flupyradifurone baits, the amount of the retrieved 0.02%, 0.01% flupyradifurone baits was significantly lower than that of the control. Therefore, sulfoxaflor, flupyradifurone, and triflumezopyrim are nonrepellent when combined with a carrier. However, the retrieval rate of 0.02% and 0.01% flupyradifurone bait decreased. Hesselbach and Scheiner [43] reported that 8.3 × 10^−4^ mol/L of flupyradifurone reduced the taste and appetitive learning performance of honeybees foraging for pollen and nectar. We speculated that 0.02% and 0.01% flupyradifurone baits may have reduced the taste and appetitive behavior of the fire ant workers, which resulted in the low retrieval rate; however, further studies are required to verify such a speculation.

Our results showed that the mortality of the fire ants treated with sulfoxaflor, flupyradifurone, and triflumezopyrim gradually increased, indicating that these baits exhibit a delayed action on fire ants, which gradually takes effect over a period of 7–14 days. Such a finding is similar to the slow effect of indoxacarb baits at extremely low concentrations [14,29,31].

Moreover, sulfoxaflor, triflumezopyrim, and flupyradifurone baits were effective in transferring their toxic properties among the fire ant colonies, as evidenced by the death of all the tested castes in 14 days. However, the female alates died more slowly than the male alates and larvae. Similarly, Ning [34] found that the female alates died the slowest among the ant castes after L-ascorbic acid treatment. Reid and Klotz [44] demonstrated that concentrations and types of insecticides affect the speed of toxicity transfer among ant colonies. Moreover, food intake, body weight, and detoxification capability may also affect the survival of different ant castes. Xiong [45] reported that the enzyme activity of female and male alates and larvae are not different. The heavier body weight and lesser food intake of the female alates during the horizontal transfer of the baits may have resulted in their longer survival time than the other castes after exposure to the sulfoxaflor, flupyradifurone, and triflumezopyrim baits.

The walking, climbing, and arrest abilities of fire ants are important for foraging and competition [46,47]. Sulfoxaflor, flupyradifurone, and triflumezopyrim baits, especially at higher concentrations, inhibited the walking, climbing, and arrest abilities of the fire ants because neonicotinoids disrupt the central nervous system of insects, causing paralysis and death [48]. The higher the concentration of baits, the stronger the inhibitory effect.

The field trials revealed that the efficacy of the treatment baits in controlling fire ant populations increased as the concentration of the baits around the mounds increased. Specifically, 0.05% sulfoxaflor, flupyradifurone, and triflumezopyrim baits had excellent potential in fire ant control and are suitable for wide area applications. However, our study has a limitation in that the experiments on the effects of treatments on the behavior of fire ants in this study were simple and the drugs used were not technical material.

When the effects of the application dosage of the baits were tested, it was found that 0.05% sulfoxaflor and triflumezopyrim baits were as effective as commercially available indoxacarb and fipronil products for fire ant control. Since cross-resistance has not been reported between sulfoxaflor, triflumezopyrim, indoxacarb, and hydramethylnon [49,50], it can be concluded that sulfoxaflor and triflumezopyrim are suitable ingredients for fire ant baits.

Cost is a factor that must be considered when performing chemical fire ant control. In this study, the market prices of 22% sulfoxaflor SC and 10% triflumezopyrim SC were 0.46 CNY/mL and 1.18 CNY/mL, respectively, whereas those of acetone, lard, and the corn grit carrier were 0.15 CNY/mL, 0.05 CNY/g, and 14.0 CNY/kg, respectively. The cost of 0.05% sulfoxaflor and triflumezopyrim baits was 1.6 CNY/mound, whereas that of the 0.1% indoxacarb bait was approximately 2.5 CNY/mound. This means even after considering the cost of manufacturing the bait carrier, the cost of the baits discussed in this article is lower than those commonly used on the market.

In this study, the efficacy of varying concentrations of prospective ingredients—sulfoxaflor, flupyradifurone, and triflumezopyrim—for fire ant baits was evaluated. Sulfoxaflor, flupyradifurone, and triflumezopyrim were nonrepellent when combined with a carrier, destroyed the walking, climbing, and arrest abilities of the fire ants, causing the death of the various castes within a fire ant colony. Particularly, 0.05% sulfoxaflor and triflumezopyrim baits are favorable competitive products for fire ant control in terms of price and efficacy. Additional effects of sulfoxaflor and triflumezopyrim on fire ants need to be studied further. This means that the cost of the agents discussed in this article is lower than those commonly used on the market.

## Figures and Tables

**Table 1:** Mortality of fire ant colonies after sulfoxaflor, flupyradifurone, and triflumezopyrim bait treatment in laboratory condition.

Bait Ingredient and Concentration	Mortality after 7 d of Treatment (%)	Mortality after 14 d of Treatment (%)	Mortality after 21 d of Treatment (%)	Mortality after 28 d of Treatment (%)
0.005% sulfoxaflor	12.67 ± 1.14 d	21.50 ± 2.04 c	32.33 ± 1.76 d	51.67 ± 1.45 d
0.01% sulfoxaflor	22.50 ± 1.38 c	31.00 ± 2.31 c	52.17 ± 2.59 c	65.33 ± 2.59 c
0.02% sulfoxaflor	33.50 ± 1.88 b	53.17 ± 3.38 b	61.83 ± 2.54 b	83.17 ± 1.89 b
0.05% sulfoxaflor	65.58 ± 4.60 a	91.83 ± 2.33 a	100.00 ± 0.00 a	100.00 ± 0.00 a
0.1% indoxacarb (positive control)	70.83 ± 4.56 a	96.67 ± 1.74 a	100.00 ± 0.00 a	100.00 ± 0.00 a
Corn grit carrier (negative control)	0.00 ± 0.00 e	3.67 ± 1.43 d	12.00 ± 1.59 e	15.00 ± 1.59 e
0.005% flupyradifurone	5.83 ± 1.17 d	15.00 ± 1.53 e	29.17 ± 2.21 c	43.33 ± 2.67 c
0.01% flupyradifurone	15.83 ± 1.87 c	24.83 ± 1.74 d	35.50 ± 2.01 c	47.33 ± 3.39 c
0.02% flupyradifurone	25.17 ± 3.36 b	36.5 ± 2.56 c	54.83 ± 1.51 b	62.50 ± 3.21 b
0.05% flupyradifurone	28.67 ± 3.99 b	46.33 ± 2.12 b	57.17 ± 1.58 b	70.83 ± 1.51 b
0.1% indoxacarb (positive control)	70.83 ± 4.56 a	96.67 ± 1.74 a	100.00 ± 0.00 a	100.00 ± 0.00 a
Corn grit carrier (negative control)	0.00 ± 0.00 e	3.67 ± 1.43 f	12.00 ± 1.59 d	15.00 ± 1.59 d
0.005% triflumezopyrim	10.17 ± 1.28 e	18.50 ± 1.48 d	31.50 ± 1.82 e	47.50 ± 1.80 e
0.01% triflumezopyrim	20.17 ± 0.79 d	28.83 ± 1.28 c	42.17 ± 2.32 d	62.00 ± 1.32 d
0.02% triflumezopyrim	33.50 ± 1.88 c	53.17 ± 3.38 b	61.83 ± 2.54 c	80.50 ± 1.18 c
0.05% triflumezopyrim	40.17 ± 1.30 b	53.50 ± 2.08 b	74.00 ± 1.46 b	88.50 ± 2.78 b
0.1% indoxacarb (positive control)	70.83 ± 4.56 a	96.67 ± 1.74 a	100.00 ± 0.00 a	100.00 ± 0.00 a
Corn grit carrier (negative control)	0.00 ± 0.00 f	3.67 ± 1.43 e	12.00 ± 1.59 f	15.00 ± 1.59 f

The data are the mean ± standard error. Identical letters in a column indicate no significant difference (*p* > 0.05). For sulfoxaflor and flupyradifurone, a Kruskal–Wallis test was used for 7, 21, and 28 d; Tukey’s test was used for 14 d. For sulfoxaflor and flupyradifurone, a Kruskal–Wallis test was used for 7 d and 28 d; Tukey’s test was used for 14 d and 21 d.

**Table 2:** Impact of sulfoxaflor at different concentrations on the behavior of fire ant workers.

Bait Ingredient and Concentration	Walking Rate (%)	Climbing Rate (%)	Arrest Rate (%)
0.005% sulfoxaflor	76.50 ± 2.06 a	74.00 ± 2.27 a	67.75 ± 2.86 a
0.01% sulfoxaflor	71.50 ± 3.66 a	72.00 ± 3.00 a	52.00 ± 2.39 b
0.02% sulfoxaflor	59.50 ± 3.38 b	60.50 ± 3.40 b	44.50 ± 3.64 b
0.05% sulfoxaflor	47.75 ± 5.16 c	48.75 ± 4.52 c	37.00 ± 2.95 b
Corn grit carrier (negative control)	98.25 ± 0.80 d	98.50 ± 0.82 d	99.00 ± 0.53 c

The data are the mean ± standard error. Identical letters in a column indicate no significant difference (*p* > 0.05) by Tukey’s test and the Kruskal–Wallis test. The Kruskal–Wallis test was used for arrest rate; Tukey’s test was used for walking rate and climbing rate.

**Table 3:** Impact of flupyradifurone at different concentrations on the behavior of fire ant workers.

Bait Ingredient and Concentration	Walking Rate (%)	Climbing Rate (%)	Arrest Rate (%)
0.005% flupyradifurone	83.00 ± 2.07 a	77.75 ± 3.17 a	78.00 ± 2.56 a
0.01% flupyradifurone	69.00 ± 2.80 a	69.75 ± 2.28 a	68.25 ± 2.37 a
0.02% flupyradifurone	73.50 ± 4.27 a	71.25 ± 2.78 a	54.75 ± 2.33 b
0.05% flupyradifurone	62.75 ± 4.09 a	72.25 ± 4.88 a	54.00 ± 2.20 b
Corn grit carrier (negative control)	97.50 ± 0.82 b	98.25 ± 0.70 b	99.00 ± 0.53 c

The data are the mean ± standard error. Identical letters in a column indicate no significant difference (*p* > 0.05) by Tukey’s test and the Kruskal–Wallis test. The Kruskal–Wallis test was used for climbing rate and arrest rate; Tukey’s test was used for walking rate.

**Table 4:** Impact of triflumezopyrim at different concentrations on the behavior of fire ant workers.

Bait Ingredient and Concentration	Walking Rate (%)	Climbing Rate (%)	Arrest Rate (%)
0.005% triflumezopyrim	79.00 ± 2.33 a	80.75 ± 2.95 a	79.50 ± 1.76 a
0.01% triflumezopyrim	71.25 ± 2.33 a	75.25 ± 3.18 a	60.75 ± 2.23 b
0.02% triflumezopyrim	63.00 ± 4.58 a	74.75 ± 3.50 a	53.75 ± 3.39 b
0.05% triflumezopyrim	56.75 ± 2.83 a	66.50 ± 4.08 a	47.25 ± 3.23 b
Corn grit carrier (negative control)	98.25 ± 2.83 d	98.50 ± 0.82 b	99.25 ± 0.37 c

The data are the mean ± standard error. Identical letters in a column indicate no significant difference (*p* > 0.05) by Tukey’s test and the Kruskal–Wallis test. The Kruskal–Wallis test was used for walking rate and arrest rate; Tukey’s test was used for climbing rate.

**Table 5:** The food discovery time and weight removed by fire ants on sulfoxaflor, flupyradifurone, and triflumezopyrim baits under laboratory conditions.

Concentration	Sulfoxaflor		Flupyradifurone		Triflumezopyrim	
	Discovery Time (s)	Bait Removed (mg)	Discovery Time (s)	Bait Removed (mg)	Discovery Time (s)	Bait Removed (mg)
0.02%	75.3 ± 10.8 a	121.1 ± 30.8 a	254.9 ± 100.0 a	13.3 ± 8.9 a	187.1 ± 58.0 a	86.7 ± 24.4 a
0.01%	110.7 ± 69.9 a	131.1 ± 41.7 a	211.4 ± 63.6 a	14.4 ± 9.3 a	126.8 ± 25.9 a	120.0 ± 35.7 a
0.005%	69.3 ± 23.2 a	187.8 ± 27.6 a	131.1 ± 38.6 a	76.7 ± 15.3 b	168.1 ± 52.4 a	110.5 ± 20.7 a
Corn grit carrier	40.4 ± 10.0 a	180.0 ± 28.0 a	141.2 ± 48.6 a	137.8 ± 44.1 b	109.6 ± 28.2 a	163.3 ± 40.5 a

The data are the mean ± standard error. Identical letters in a column indicate no significant difference (*p* > 0.05) by Tukey’s test for sulfoxaflor and triflumezopyrim, and the Kruskal–Wallis test and the Mann–Whitney test for flupyradifurone.

**Table 6:** The food discovery time and weight removed by fire ants on sulfoxaflor, flupyradifurone, and triflumezopyrim baits under field conditions.

Concentration	Sulfoxaflor		Flupyradifurone		Triflumezopyrim	
	Discovery Time (s)	Bait Removed (mg)	Discovery Time (s)	Bait Removed (mg)	Discovery Time (s)	Bait Removed (mg)
0.02%	61.7 ± 13.6 a	658.3 ± 249.9 ab	56.8 ± 8.5 a	476.7 ± 228.0 a	53.0 ± 13.7 a	241.7 ± 77.8 a
0.01%	48.3 ± 9.1 a	895.0 ± 335.3 ab	66.5 ± 12.5 a	493.3 ± 104.8 a	55.5 ± 9.7 a	250.0 ± 63.7 a
0.005%	64.5 ± 13.3 a	985.0 ± 389.7 ab	52.8 ± 12.7 a	370.0 ± 94.0 a	51.7 ± 12.5 a	345.0 ± 118.0 a
0.1% indoxacarb	41.5 ± 10.2 a	1088.3 ± 375.8 b	40.2 ± 11.9 a	188.3 ± 58.8 a	44.7 ± 11.1 a	708.3 ± 285.1 a
Corn grit carrier	48.0 ± 9.4 a	285.0 ± 60.75 a	62.2 ± 12.3 a	205.0 ± 33.1 a	55.8 ± 9.4 a	715.0 ± 203.0 a

The data are the mean ± standard error. Identical letters in a column indicate no significant difference (*p* > 0.05) by Tukey’s test for discovery time and the Kruskal–Wallis test for bait removed.

**Table 7:** Control effects of fire ant colonies 35 days post-treatment with sulfoxaflor, flupyradifurone, and triflumezopyrim toxic bait.

Bait Ingredient and Concentration	Mortality of Fire Ant Mounds (%)	Control Effect of Fire Ant Colonies (%)	Average Activity Level of Fire Ant Colonies
0.005% sulfoxaflor	29.17 ± 4.17 d	50.00 ± 0.00 c	1.37 ± 0.03 d
0.01% sulfoxaflor	45.83 ± 4.17 c	57.73 ± 5.43 c	1.13 ± 0.15 c
0.02% sulfoxaflor	70.83 ± 7.22 b	78.2 ± 0.90 b	0.57 ± 0.03 b
0.05% sulfoxaflor	91.67 ± 4.17 a	92.27 ± 1.37 a	0.23 ± 0.07 a
0.1% indoxacarb (positive control)	100.00 ± 0.00 a	100.00 ± 0.00 a	0.00 ± 0.00 a
Corn grit carrier (negative control)	0.00 ± 0.00 e	0.00 ± 0.00 d	2.70 ± 0.10 e
0.005% flupyradifurone	12.50 ± 0.00 d	24.87 ± 2.43 c	2.00 ± 0.00 d
0.01% flupyradifurone	20.83 ± 4.17 d	38.80 ± 4.60 b	1.63 ± 0.08 c
0.02% flupyradifurone	37.50 ± 0.00 c	46.83 ± 1.59 b	1.43 ± 0.03 bc
0.05% flupyradifurone	54.17 ± 4.17 b	49.83 ± 2.74 b	1.36 ± 0.03 b
0.1% indoxacarb (positive control)	100 ± 0.00 a	100.00 ± 0.00 a	0.00 ± 0.00 a
Corn grit carrier (negative control)	0.00 ± 0.00 e	0.00 ± 0.00 d	2.70 ± 0.10 e
0.005% triflumezopyrim	37.50 ± 7.22 c	31.37 ± 2.23 c	1.83 ± 0.12 c
0.01% triflumezopyrim	62.50 ± 7.22 b	45.17 ± 7.04 c	1.47 ± 0.20 c
0.02% triflumezopyrim	66.67 ± 4.17 b	65.43 ± 3.78 b	0.93 ± 0.07 b
0.05% triflumezopyrim	83.33 ± 4.17 ab	73.33 ± 4.27 b	0.73 ± 0.12 b
0.1% indoxacarb (positive control)	100.00 ± 0.00 a	100.00 ± 0.00 a	0.00 ± 0.00 a
Corn grit carrier (negative control)	0.00 ± 0.00 d	0.00 ± 0.00 d	2.70 ± 0.10 d

The data are the mean ± standard error. Identical letters in a column indicate no significant difference (*p* > 0.05). For mortality of fire ant mounds, the Kruskal–Wallis test was used for sulfoxaflor and flupyradifurone, and Tukey’s test was used for triflumezopyrim. For the control effect of fire ant colonies, the Kruskal–Wallis test was applied. For the average activity level of fire ant colonies, Tukey’s test was applied for sulfoxaflor and triflumezopyrim, and the Kruskal–Wallis test was used for flupyradifurone.

**Table 8:** Reduction rates of fire ant workers post-treatment with sulfoxaflor, flupyradifurone, and triflumezopyrim toxic bait.

Bait Ingredient and Concentration	Reduction Rate of Forging Workers after Corresponding Days of Treatment (%)				
	7 d	14 d	21 d	28 d	35 d
0.005% sulfoxaflor	−33.2 ± 24.4 b	−68.1 ± 19.9 b	13.0 ± 15.0 ab	41.0 ± 10.6 ab	33.4 ± 8.8 b
0.01% sulfoxaflor	−20.7 ± 25.3 b	25.0 ± 8.9 ab	12.3 ± 11.1 ab	36.2 ± 7.0 ab	33.4 ± 8.0 b
0.02% sulfoxaflor	27.4 ± 16.0 ab	7.9 ± 15.6 b	21.3 ± 16.8 ab	49.0 ± 15.4 ab	36.0 ± 17.2 ab
0.05% sulfoxaflor	39.1 ± 13.2 ab	43.9 ± 3.3 a	54.0 ± 12.0 a	62.5 ± 7.7 a	59.5 ± 5.3 ab
0.1% indoxacarb (positive control)	97.6 ± 1.1 a	68.3 ± 10.0 a	56.3 ± 8.4 a	82.2 ± 2.6 a	82.8 ± 4.9 a
Corn grit carrier (negative control)	3.8 ± 21.7 b	2.8 ± 5.0 b	8.5 ± 19.9 b	20.3 ± 12.5 b	22.0 ± 11.0 b
0.005% flupyradifurone	63.5 ± 5.8 ab	45.0 ± 7.1 a	56.4 ± 6.0 a	46.9 ± 8.5 ab	63.4 ± 6.3 a
0.01% flupyradifurone	41.5 ± 12.1 ab	55.5 ± 9.8 a	66.1 ± 9.1 a	65.4 ± 8.3 a	72.6 ± 2.4 a
0.02% flupyradifurone	27.5 ± 19.4 b	46.6 ± 2.6 a	62.7 ± 7.4 a	46.8 ± 8.6 ab	57.8 ± 8.0 a
0.05% flupyradifurone	35.1 ± 14.1 ab	55.2 ± 8.0 a	55.3 ± 6.7 a	55.5 ± 11.3 a	66.6 ± 6.6 a
0.1% indoxacarb (positive control)	97.6 ± 1.1 a	68.3 ± 10.0 a	56.3 ± 8.4 a	82.2 ± 2.6 a	82.8 ± 4.9 a
Corn grit carrier (negative control)	3.8 ± 21.7 b	2.8 ± 5.0 b	8.5 ± 19.9 b	20.3 ± 12.5 b	22.0 ± 11.0 b
0.005% triflumezopyrim	31.4 ± 4.7 bc	50.4 ± 3.1 a	54.9 ± 10.7 a	73.0 ± 1.6 a	64.5 ± 3.0 a
0.01% triflumezopyrim	45.5 ± 8.1 bc	52.0 ± 8.1 a	57.9 ± 9.1 a	71.8 ± 5.4 a	67.5 ± 3.7 a
0.02% triflumezopyrim	71.6 ± 5.7 ab	69.5 ± 6.0 a	50.1 ± 14.4 a	80.6 ± 3.4 a	76.1 ± 2.4 a
0.05% triflumezopyrim	43.7 ± 8.3 bc	79.0 ± 2.4 a	76.3 ± 1.2 a	81.7 ± 2.5 a	74.2 ± 2.4 a
0.1% indoxacarb (positive control)	97.6 ± 1.1 a	68.3 ± 10.0 a	56.3 ± 8.4 a	82.2 ± 2.6 a	82.8 ± 4.9 a
Corn grit carrier (negative control)	3.8 ± 21.7 c	2.8 ± 5.0 b	8.5 ± 20.0 b	20.3 ± 12.5 b	22.0 ± 11.0 b

The data are the mean ± standard error. Identical letters in a column indicate no significant difference (*p* > 0.05). For sulfoxaflor and flupyradifurone, Tukey’s test was applied for 35 d, and the Kruskal–Wallis test was applied for triflumezopyrim.

**Table 9:** Comprehensive control effect of fire ant colonies 35 days post-treatment with sulfoxaflor, flupyradifurone, and triflumezopyrim toxic bait.

Bait Ingredient and Concentration	Comprehensive Control Effect of Fire Ant Colonies (%)
0.005% sulfoxaflor	43.43 ± 1.69 efg
0.01% sulfoxaflor	49.30 ± 5.27 ef
0.02% sulfoxaflor	67.55 ± 4.47 c
0.05% sulfoxaflor	86.43 ± 0.64 ab
0.005% flupyradifurone	28.86 ± 1.56 g
0.01% flupyradifurone	40.17 ± 2.62 efg
0.02% flupyradifurone	46.24 ± 1.08 ef
0.05% flupyradifurone	54.49 ± 3.83 def
0.005% triflumezopyrim	39.84 ± 1.64 fg
0.01% triflumezopyrim	54.84 ± 4.52 de
0.02% triflumezopyrim	67.94 ± 2.16 cd
0.05% triflumezopyrim	77.18 ± 2.75 bc
0.1% indoxacarb (positive control)	96.09 ± 0.78 a
Corn grit carrier (negative control)	2.67 ± 1.55 h

The data are the mean ± standard error. Identical letters in a column indicate no significant difference by Tukey’s test (*p* > 0.05).

**Table 10:** The mortality of fire ant mounds and reduction of foraging workers after broadcasting 0.05% sulfoxaflor, flupyradifurone, and triflumezopyrim baits.

Treatments	Mortality of Mounds after Corresponding Days of Treatment (%)		Reduction Rate of Forging Workers after Corresponding Days of Treatment (%)	
	14 d	28 d	14 d	28 d
0.05% sulfoxaflor 1.5 times application dosage	71.82 ± 0.91 a	81.21 ± 0.61a	38.00 ± 1.56 abc	71.85 ± 1.12 ab
0.05% sulfoxaflor 2.0 times application dosage	66.67 ± 0.00 a	84.44 ± 1.11a	58.40 ± 0.30 a	80.96 ± 0.62 a
0.05% flupyradifurone 1.5 times application dosage	27.22 ± 4.57 e	52.08 ± 2.98 c	29.80 ± 3.01 bcd	62.30 ± 5.58 bc
0.05% flupyradifurone 2.0 times application dosage	35.24 ± 4.01 de	52.80 ± 1.41 c	41.34 ± 5.55 abc	45.43 ± 4.07 d
0.05% triflumezopyrim 1.5 times application dosage	41.39 ± 2.83 cd	72.32 ± 1.68 b	19.94 ± 7.85 cd	41.03 ± 3.22 d
0.05% triflumezopyrim 2.0 times application dosage	53.33 ± 3.33 bc	82.50 ± 2.50 a	34.46 ± 6.77 bc	62.20 ± 0.66 bc
0.1% indoxacarb bait (positive control)	66.12 ± 1.43 ab	83.51 ± 1.71 a	44.67 ± 4.78 ab	53.71 ± 0.57 cd
Corn grit carrier (negative control)	0.00 ± 0.00 f	0.00 ± 0.00 d	11.35 ± 0.70 d	12.57 ± 1.04 e

The data are the mean ± standard error. Identical letters in a column indicate no significant difference (*p* > 0.05) by Tukey’s test.

## Data Availability

The raw data supporting the conclusions of this article will be made available by the authors on request.
